# Effects and Mechanisms of Total Flavonoids from *Blumea balsamifera* (L.) DC. on Skin Wound in Rats

**DOI:** 10.3390/ijms18122766

**Published:** 2017-12-19

**Authors:** Yuxin Pang, Yan Zhang, Luqi Huang, Luofeng Xu, Kai Wang, Dan Wang, Lingliang Guan, Yingbo Zhang, Fulai Yu, Zhenxia Chen, Xiaoli Xie

**Affiliations:** 1 National Resource Center for Chinese Materia Medica, China Academy of Chinese Medical Sciences, Beijing 100700, China; yxpang@catas.cn (Y.P.); zhangyan8669@126.com (Y.Z.); 2 Center for Post-Doctoral Research, China Academy of Chinese Medical Sciences, Beijing 100700, China; 3 Tropical Crops Genetic Resources Institute, Chinese Academy of Tropical Agricultural Sciences, Danzhou 571737, China; x1531298865@126.com (L.X.); jimojijie29@163.com (K.W.); wang_dan1414@163.com (D.W.); gllgirl123@163.com (L.G.); zhangyingbo1984@catas.cn (Y.Z.); flyu@catas.cn (F.Y.); hnchenzhenxia@126.com (Z.C.); xiexiaoli198883@163.com (X.X.); 4 Hainan Provincial Engineering Research Center for Blumea Balsamifera, Danzhou 571737, China

**Keywords:** *Blumea balsamifera* (L.) DC., total flavonoids, skin wound, VEGF, TGF-*β*_1_

## Abstract

Chinese herbal medicine (CHM) evolved through thousands of years of practice and was popular not only among the Chinese population, but also most countries in the world. *Blumea balsamifera* (L.) DC. as a traditional treatment for wound healing in Li Nationality Medicine has a long history of nearly 2000 years. This study was to evaluate the effects of total flavonoids from *Blumea balsamifera* (L.) DC. on skin excisional wound on the back of Sprague-Dawley rats, reveal its chemical constitution, and postulate its action mechanism. The rats were divided into five groups and the model groups were treated with 30% glycerol, the positive control groups with Jing Wan Hong (JWH) ointment, and three treatment groups with high dose (2.52 g·kg^−1^), medium dose (1.26 g·kg^−1^), and low dose (0.63 g·kg^−1^) of total flavonoids from *B. balsamifera*. During 10 consecutive days of treatment, the therapeutic effects of rates were evaluated. On day 1, day 3, day 5, day 7, and day 10 after treatment, skin samples were taken from all the rats for further study. Significant increases of granulation tissue, fibroblast, and capillary vessel proliferation were observed at day 7 in the high dose and positive control groups, compared with the model group, with the method of 4% paraformaldehyde for histopathological examination and immunofluorescence staining. To reveal the action mechanisms of total flavonoids on wound healing, the levels of CD68, vascular endothelial growth factor (VEGF), transforming growth factor-*β*_1_ (TGF-*β*_1_), and hydroxyproline were measured at different days. Results showed that total flavonoids had significant effects on rat skin excisional wound healing compared with controls, especially high dose ones (*p* < 0.05). Furthermore, the total flavonoid extract was investigated phytochemically, and twenty-seven compounds were identified from the total flavonoid sample by ultra-high-performance liquid chromatography coupled with quadrupole time-of-flight mass spectrometry/diode array detector (UPLC-Q-TOF-MS/DAD), including 16 flavonoid aglucons, five flavonoid glycosides (main peaks in chromatogram), five chlorogenic acid analogs, and 1 coumarin. Reports show that flavonoid glycoside possesses therapeutic effects of curing wounds by inducing neovascularization, and chlorogenic acid also has anti-inflammatory and wound healing activities; we postulated that all the ingredients in total flavonoids sample maybe exert a synergetic effect on wound curing. Accompanied with detection of four growth factors, the upregulation of these key growth factors may be the mechanism of therapeutic activities of total flavonoids. The present study confirmed undoubtedly that flavonoids were the main active constituents that contribute to excisional wound healing, and suggested its action mechanism of improving expression levels of growth factors at different healing phases.

## 1. Introduction

Acute wound healing proceeds through four stages: inflammation response, migration, proliferation, and tissue remodeling [1]. When the skin is injured, the normal healing response begins. During inflammation and initial stages of wound healing, the process of repair is largely mediated by cytokines or growth factors such as tumor necrosis factor alpha (TNF-*α*), transforming growth factor-beta (TGF-*β*) [2,3], platelet derived growth factor (PDGF), and vascular endothelial growth factor (VEGF) that orchestrate the manifold cellular activities [4,5,6]. Simultaneously, specialized cells move to the wound site. In the inflammatory phase, polymorphonuclear neutrophilic leukocytes and macrophages appear around the wounds. Macrophages, as the principal phagocytic cells in wound repair, provide an effective local barrier against bacterial invasion and wound debridement. Macrophages can also induce production of TNF-*α,* TGF-*β*, CD68, and other factors [7,8,9]. The proliferative phase is characterized by angiogenesis, collagen deposition, epithelialization, and wound contraction [10]. In this phase, fibroblasts act as the principal cells responsible for collagen deposition to form a new, provisional extracellular matrix [11]. Collagen is the most abundant protein, accounting for 30% of the total protein in the human body [12]. Collagen contains substantial amounts of hydroxyproline, which is used as a biochemical marker for tissue collagen [13]. The aforementioned contents resulted in our selection of four growth factors of TGF-*β*, VEGF, CD68, and hydroxyproline at different stages as biomarkers to elucidate the wound healing mechanism.

*Blumea balsamifera* (L.) DC., belongs to *Blumea*, Compositae, widely distributed in Hainan, Guizhou, Yunnan, Guangdong, and Taiwan provinces in China [14,15]. Its leaves, twigs, and roots were widely used for many diseases, for instance, rheumatism, dermatitis, beriberi, and lumbago, and especially for treatment of snake bites and bruises in some ancient Chinese minorities such as Li, Miao, and Zhuang [16]. Recent pharmacological studies have showed that *B. balsamifera* possesses a broad spectrum of pharmacological activities such as coagulation [17], antibacterial, free radical scavenging [18], antioxidant [19], and anticancer [20,21], while few studies have investigated and reported on the injure curing effects of this plant. Phytochemical analysis revealed that *B. balsamifera* leaves contained considerable amounts of flavonoids [22], and related literature studies also show that many flavonoids possess direct or indirect wound healing effects, such as soy isoflavone on scalded mice [23], and corylin on human fibroblast cells in an in vitro model of wound healing [24].

However, flavonoids, once discarded in residue after l-borneol was distilled, were proved to be the main ingredients in *B. balsamifera*, and may contribute to many traditional usages of this herb. As part of our ongoing search for skin recovery candidates, we investigated the chemical constituents of total flavonoids from *B. balsamifera* using UPLC-Q-TOF-MS/DAD, evaluated the its wound curing effects, and elucidated the underlying mechanisms of total flavonoids in the process of wound healing.

## 2. Results

### 2.1. Content and Identification of Total Flavonoids

The typical calibration plot conducted with rutin as standard resulted in the regression equation: *y* = 5.1907*x* − 0.0098, and accordingly, the content of total flavonoids was deduced to be 81.1% (Figures S1 and S2). From literature reported [22], *B. balsamifera* is rich in flavonoids, and nearly forty flavonoid analogs were isolated and identified from this plant, including structure types of flavonoid glycoside and aglucon.

**Figure 1:**
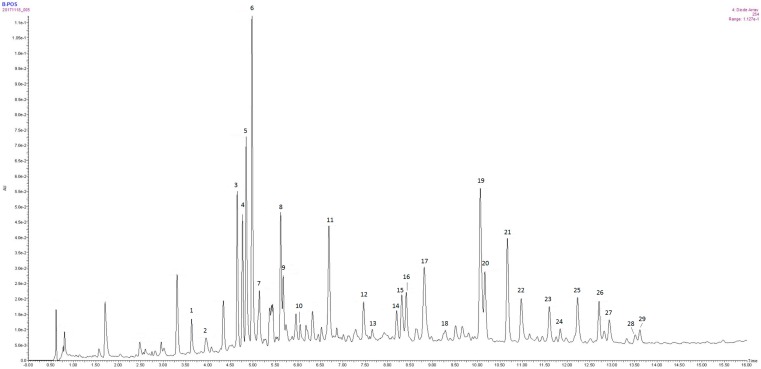
UPLC chromatograms at 254 nm of total flavonoids sample in positive ion modes analyzed by UPLC-Q-TOF/MS/DAD.

Investigation of the chemical constitution of total flavonoids recorded with UPLC-Q-TOF-MS/DAD led to the identification of 27 compounds (two unidentified of 29 peaks) (Figure 1). The base peak intensity (BPI) and ultra-high-performance liquid chromatography (UPLC) chromatogram at 254 nm of total flavonoids of *Blumea balsamifera* (L.) DC. with positive ion electrospray ionization (ESI) and negative ESI were recorded (as shown in Figure S3). The measured MS data exhibited high consistency with theoretical values, with deviation limited within 5 ppm, which provide valuable information for the determination of constituents. Twenty-seven compounds, including 21 flavonoids, were tentatively confirmed on the basis of their retention behaviors (Figure S4), accurate molecular weight, and MS^E^ fragment data, and comparison with closely related substances reported in literature (chemical structures are shown in Figure S5, corresponding quasi-molecular ions are listed in Table S1).

The peaks of 3, 4, 5, 6, and 7, ascribed to the chromatogram, accounted for a large proportion and were identified to be the same structure type of flavonoid glycoside (Figure S5), which indicated flavonoid glycosides may be the main effective ingredients of total flavonoids based on related literature [25].

### 2.2. Effects of Different Doses of Total Flavonoids on Wound Healing Rates in Rats

**Figure 2:**
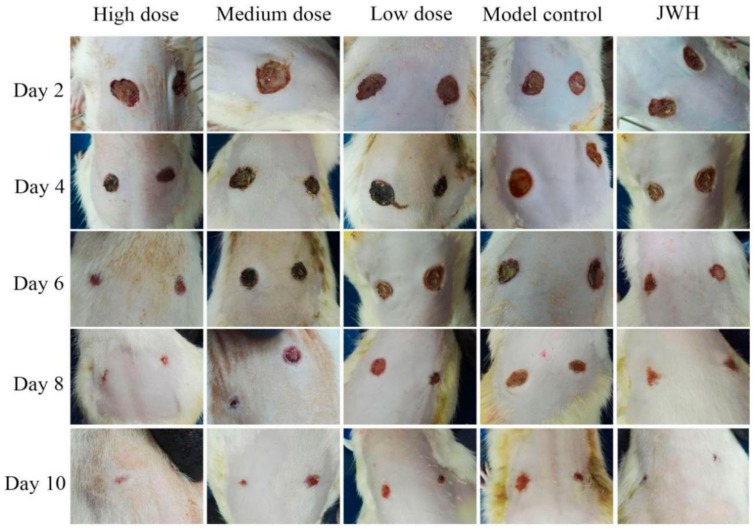
Recovery of wounds at different times. JWH, Jing Wan Hong.

Treatments were carried out after surgery, and photos of wounds were taken on indicated days. All rats lost weight on the first day after excision, while two days later, their weights steadily increased, and the wound tissues were ruddy and edema disappeared (Figure 2). There was no significant difference between all groups. On day 4, there were no wound infections except the model control, and all rats in the different groups developed granulation tissue growth and significantly reduced wound area. However, the groups with high and medium doses of total flavonoids demonstrated accelerated re-epithelization compared with the model groups. The black crusts were residue resulting from the absorption of total flavonoids. On day 6, the black crusts exfoliated in some rats of the high dose and Jing Wan Hong (JWH) groups. On day 8, the high and medium dose groups showed significantly accelerated wound contraction and closures compared with the model groups. On day 10, the percentage of wound contraction was nearly 100% in the high dose groups, and wound contraction in the other groups were also fully completed, but surprisingly, rats with total flavonoids showed better effects on the wound area and epithelization than the other groups.

**Figure 3:**
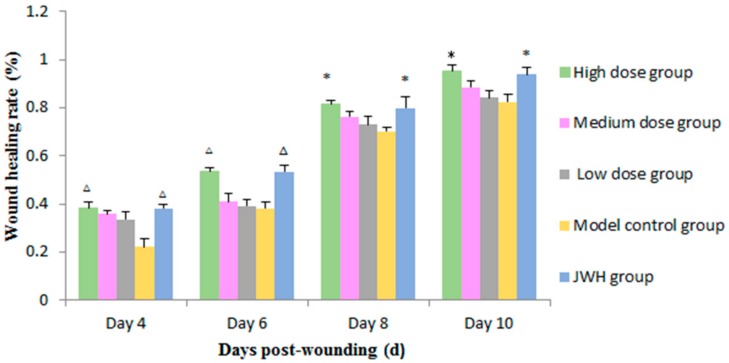
Effects of total flavonoids from *B. balsamifera* on wound healing rate of rats. Values are expressed as mean ± SD (*n* = 6) as compared to the control group, * *p* < 0.05, ^∆^ *p* < 0.01.

As shown in Figure 3 (Table S2), on day 4 and 6, the high dose and JWH groups showed significantly better effects of wound healing than the model groups (*p* < 0.01). On day 8, wound healing was obviously better in high dose groups (*p* = 0.011) and JWH groups (*p* = 0.032) than the model groups. Until day 10, the wound healing rates of high dose and JWH groups were approximately 95.0%, while the rate of model groups was lower than 85.0%, which indicated the potent efficiency of total flavonoids, especially at high doses. The rates in the medium dose and low dose groups were also higher than the model group, but not statistically significant.

### 2.3. Effects of Different Doses of Total Flavonoids on CD68 Levels on Rats

**Figure 4:**
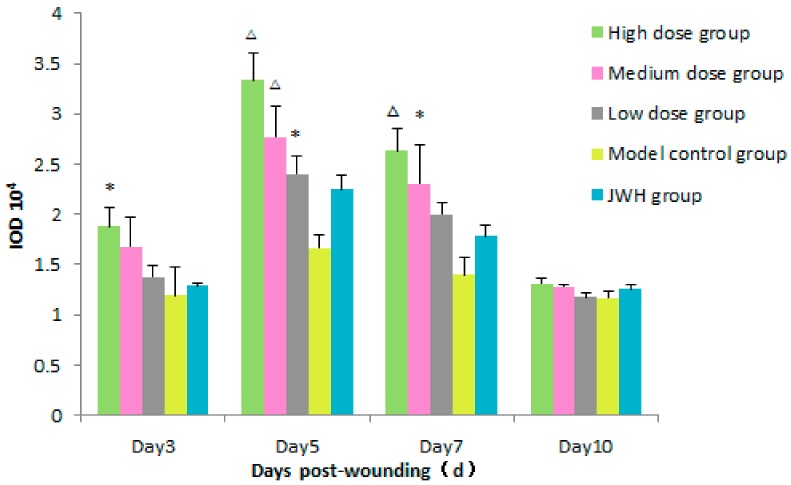
Effects of total flavonoids from *B. balsamifera* on CD68 levels in wound tissues of rats. Values are expressed as mean ± SD (*n* = 6) as compared to control group, * *p* < 0.05, ^∆^ *p* < 0.01.

CD68 is a special antigen expressed by macrophages and cells of myeloid/mononuclear lineage [9]. As shown in Figure 4 (Table S3), in each of the total flavonoids groups, the increased CD68 expression in skin wounds indicated that total flavonoids contributed much to the increasing numbers of macrophages. On day 3, the average integral optical density (IOD) of CD68 in high dose groups were higher than that of control groups (*p* < 0.05). On day 5, CD68 levels of all total flavonoids groups reached a peak (*p* = 0.005, 0.009, and 0.036, respectively). On day 7, the average IOD values of CD68 in high and medium dose groups were still higher than control (*p* = 0.003 and 0.015, respectively). However, on day 10, there were no significant differences between all groups. The changing tendency of CD68 levels indicated that macrophages were very active in the inflammatory stage of high dose treatments before day 5, and downregulated in the following days.

### 2.4. Effects of Total Flavonoids on VEGF and TGF-β_1_ of the Wound Tissues on Rats

**Figure 5:**
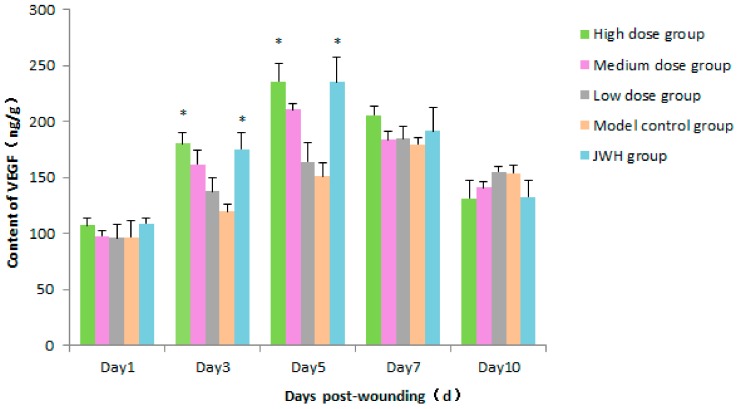
Effects of total flavonoids from *B. balsamifera* on vascular endothelial growth factor (VEGF) levels in wound tissues of rats. Values are expressed as mean ± SD (*n* = 6) as compared to the control group, * *p* < 0.05.

The expression of VEGF was able to drive the proliferation of endothelial cells and formation of new vessels. As shown in Figure 5 (Table S4), VEGF expression of total flavonoid groups increased in a dose-dependent manner (*p* < 0.05) compared to controls. Their tendencies were in an obviously descending order: high dose > medium dose > low dose. VEGF expression reached a peak in high dose, medium dose, and JWH groups on day 5. However, the peaks converted to low dose and control groups after day 7. These results also revealed that VEGF is very active in the crucial curing related stage of day 3 to 7. The peak appearance of high and medium doses ahead of time indicated their acceleration of wound curing.

**Figure 6:**
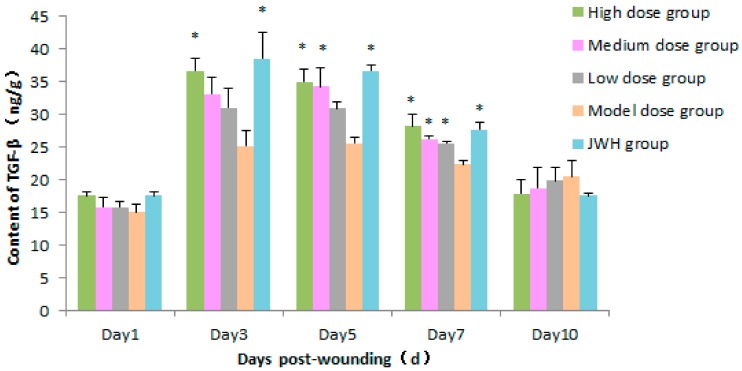
Effects of total flavonoids from *B. balsamifera* on TGF-*β*_1_ levels in wound tissues of rats. Values are expressed as mean ± SD (*n* = 6) as compared to the control group, * *p* < 0.05.

TGF-*β*_1_ could induce gathering of inflammatory cells to cuts, which is important to the curing process [26]. On day 1, there was no significant difference in TGF-*β*_1_ levels in wound tissues between each group. On day 3 and 5, total flavonoids of low dose were able to mildly stimulate the expression of TGF-*β*_1_, but its effect was obvious as compared to the model control (*p* > 0.05) (Figure 6 and Table S5). The results indicated the enrichment and proliferation of macrophages and fibroblasts in this stage. On day 7, total flavonoids still were able to promote TGF-*β*_1_ levels at all concentrations tested as compared to model control groups (*p* < 0.05 or *p* < 0.01), but the TGF-*β*_1_ levels of all groups began to drop. On day 10, TGF-*β*_1_ expression induced by wounding was markedly decreased to normal level.

### 2.5. Effects of Total Flavonoids on Hydroxyproline Level in Wound Tissues of Rats

**Figure 7:**
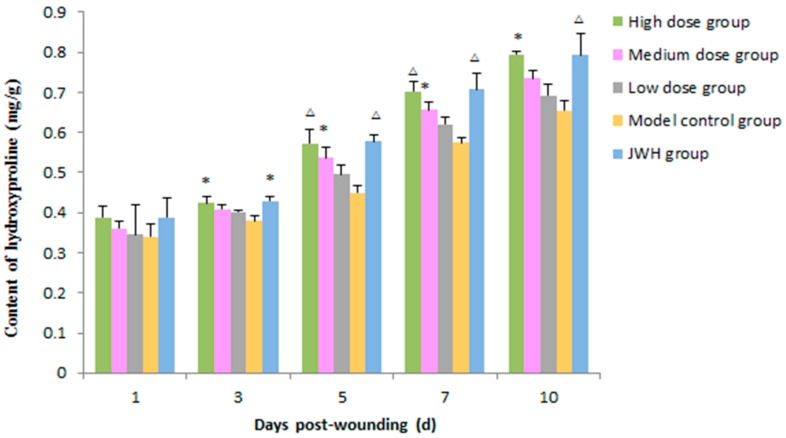
Effects of total flavonoids from *B. balsamifera* on hydroxyproline level in wound tissues of rats. Values are expressed as mean ± SD (*n* = 6) as compared to the control group, * *p* < 0.05, ^∆^ *p* < 0.01.

Hydroxyproline content is a stable parameter of collagen, which correlated with the growth of granulation tissue. On day 3 after the wound was treated, the high dose and JWH groups began to show statistically significant differences in hydroxyproline contents compared to the model control (*p* < 0.05) (Figure 7 and Table S6). Hydroxyproline expression peaked on day 10. On day 5 and 7, hydroxyproline content maintained an ascending tendency. Leading up to day 10, hydroxyproline contents in the granulation tissue of high dose and JWH groups reached a peak and were approximately 0.80 mg/g, while the contents in model groups were less than 0.65 mg/g. The contents in the medium dose and low dose groups were also higher than the model groups, but there were no significant differences in wound tissues between groups. These results revealed that the total flavonoids of *B. balsamifera* were able to promote synthesis of collagen and accelerate formation of granulation tissue in the middle and late period of wound recovery.

## 3. Discussion

Skin trauma is very prevalent in the world, research studies showed that more than one million people each year in America may acquire skin injuries, so a search for effective drugs with low side effects is desperately needed. Nowadays, Traditional Chinese Medicine (TCM) is widely used in treatments of many diseases due to its safety and minimum side effects. Within considering both the traditional uses of *B. balsamifera* for treatments of traumatic injury [16] as well as the main ingredients of flavonoids it contains, these aforementioned factors indicated the potential therapeutic effects of total flavonoids in wound healing, which led to our present ongoing study. Chemical investigation, wound healing effects, and the pharmacological mechanism of the total flavonoids were studied. In the present study, our data revealed that total flavonoids could significantly promote wound healing, improve wound contraction, and accelerate epithelialization.

Analysis of the total flavonoids extract sample with UPLC-Q-TOF-MS/DAD resulted in the identification of 27 compounds (2 unidentified of 29 peaks), including 16 flavonoid aglucons, five flavonoid glycosides, five chlorogenic acid (CQA) derivatives, and one coumarin, which indicated flavonoid derivatives as the main effective constituents. Considering the reported activity of flavonoid glycoside on wound healing [27] and the efficiency of chlorogenic acid on anti-inflammatory and wound healing [28,29], it is suggested that 16 flavonoid aglucons, five flavonoid glycosides, and five chlorogenic acid analogs may possess some joint synergetic effects in the wound curing process. This also provides a novel insight into the therapeutic effects of total flavonoids.

Wound healing was a coordinated effort of several growth factors, cytokines, and chemokines. The TGF-*β* family and VEGF family played significant roles in this process, and proper wound healing was guided by stringent regulation of these agents as well as a wound environment that favors their activity [26]. Reports show that vaccarin, a flavonoid glycoside, can induce neovascularization and accelerate wound healing by promoting the expression of CD31 levels and enhanced protein expression of p-Akt and p-Erk [27], which indicates that the flavonoid glycosides (accounting for a large proportion of chromatogram in this study) may be the main active ingredients for wound healing; together with other flavonoids and chlorogenic acids, they may contribute to joint therapeutic effects by prompting growth factors, accelerating neovascularization, and inducing tissue formation in different stages. Our data demonstrated that total flavonoids of *B. balsamifera* can promote the expression of TGF-*β*_1_ compared to control groups, while TGF-*β*_1_ plays a key role in the recruitment of additional inflammatory cells, enhancing tissue debridement of macrophages, and promoting collagen and granulation tissue formation [30,31,32]. Increased expression of TGF-*β*_1_ in *B. balsamifera* groups may be due to stimulation of the macrophages induced by total flavonoids in wounds, because earlier studies suggested that macrophages can enhance the contents of TGF-*β*_1_ [33]. CD68 antigen, as a pan-macrophage marker, can be used as an essential indicator to evaluate quantities of macrophages, and macrophages contribute greatly to inflammatory reaction of wound. Our data showed an increased expression of CD68, which suggested an increasing number of macrophages and was in accordance with the increased expression of TGF-*β*_1_.

The granulation tissue of wounds is primarily composed of fibroblasts, collagen, and new small blood vessels. Hydroxyproline, the major component of collagen, has been used as a biochemical marker for tissue collagen [34]. Our results showed that hydroxyproline content was significantly different to control. TGF-*β*_1_ is known for promoting fibroblast proliferation [35,36], therefore, it is likely that high expression of TGF-*β*_1_ resulted in an increase in hydroxyproline. VEGF can promote angiogenesis and increase vascular permeability. It gathers macrophages, fibroblasts, and some other cells around the wound site [37,38]. VEGF expression increased in total flavonoids groups compared to controls, which indicated that the total flavonoids could significantly promote wound healing not only due to increased collagen synthesis, but also due to its inducing expression of cytokines. Flavonoids in *B. balsamifera* are well known for their astringent, free radical-scavenging activity and antimicrobial properties [21,39,40]. Our present investigations further enlarged total flavonoids’ activities, and elucidated the pharmacological mechanisms in wound contraction and promotion of epithelialization.

Collectively, these results suggested that total flavonoids of *B. balsamifera* possess significant therapeutic effects on skin injuries. A phytochemical investigation uncovered the composition of total flavonoids, including twenty-one flavonoids, five chlorogenic acids, and one coumarin. We postulated their joint synergistic therapeutic mechanism as a whole agent based on measurement and analysis of several representative biomarkers, accompanied with related literature studies, which will be helpful for understanding action mechanisms of TCM and selecting candidates for clinical therapy for skin injuries in the future.

## 4. Materials and Methods

### 4.1. Plant Collection and Total Flavonoids Preparation

Leaves of *Blumea balsamifera* (L.) DC. were collected from Danzhou, Hainan, China and authenticated by Dr. Yuxin Pang, professor of the Tropical Crops Genetic Resources Institute, Chinese Academy of Tropical Agricultural Sciences. Jing Wan Hong cream was produced by Tianjin Darentang Jingwanhong Pharmaceutical Co., Ltd. (Tianjin, China). The air dried *Blumea balsamifera* (L.) DC. leaves (400 g) were extracted with 95% methanol reflux (2X3L, each for two hours). The organic solvent was evaporated in vacuo to afford a dark residue, the residue was then suspended in water and fractionated with petroleum ether three times, the water part was evaporated until there were no organic solvent odors, and then subjected to polyamide macroporous resin column chromatography, eluting with a MeOH/H_2_O gradient (0/100, 80/20, 100/0) to afford three fractions (F1–F3). The F2, eluted with 80% methanol, was evaluated for total flavonoids content of 81.1% with the method of UV-Vis spectrophotometry [41,42].

### 4.2. Establishment of Rutin Standard Curve

The total flavonoids sample was prepared (100 mg) by dissolving with 2 mL 75% methanol and 100 µL was transferred from pipette to 25 mL volumetric flask, to which a series of solvents, including 75% methanol 10 mL, 5% NaNO_2_ 1 mL, 10% Al(NO_3_)_3_ 1 mL, 4% NaOH 10 mL, and then 75% methanol, were added into the measuring flask accurately in turns.

The standard rutin (18 mg) was dissolved with 75% methanol and transferred accurately to a 10-mL measuring flask, with different volumes of rutin solution: 0.25 mL, 0.5 mL, 1 mL, 2 mL, 2.5 mL, and 3.35 mL were transferred to 10-mL measuring flasks respectively with solvents added the same as the total flavonoids to form different concentrations of standard rutin samples. All the different solutions of rutin had their absorbance recorded via UV-Vis spectrophotometer at the maximum absorbance wavelength of 500 nm (Figure S1), and a standard curve was formed with concentrations listed on the *X*-axis, and absorbance listed on the *Y*-axis (Figure S2).

### 4.3. Qualitative Characteristics of Chemical Constituents of Total Flavonoids Extract

Identification of chemical constituents in the total flavonoids extract was performed by UPLC-Q-TOF/MS/DAD analysis and the UPLC-MS spectra of samples were acquired in positive and negative modes. The optimized UPLC-MS condition is shown in the Supplementary Materials.

### 4.4. Animals

A total of 150 healthy Sprague-Dawley (SD) rats of specific pathogen-free (SPF) grade, weighing 200–240 g, were supplied by Changsha Tianqin Biotech Ltd. (Certificate of quality No. SCXK (xiang) 2014-0011, date: 4 September 2014 to 4 September 2019), Changsha, China. All rats were maintained under 24 °C, with 55–65% humidity and a 12-h light/dark cycle in the Laboratory of Tropical Medicinal Plants Resources, Tropical Crops Genetic Resources Institute, Chinese Academy of Tropical Agricultural Sciences, Danzhou, China before use. The handling and care of the rats abided by the National Institutes of Health (NIH) guidelines for animal research, and all experimental protocols were approved by the National Research Institute for Child Health and Development Animal Care and Use Committee (Permit Number: S24018). All animal experiments were performed according to these guidelines. Many efforts were made to minimize the suffering of the rats.

### 4.5. Animal Modeling and Drug Treatments

Full-thickness skin excision wounds of 1 cm diameter were created by removing the whole dorsal skin layer on both sides of the backbone of rats. All rats were randomly divided into five groups, with 30 rats per group, and treated with the random number table method: the model group treated with 30% glycerol solution, the positive control treated with JWH cream, and three total flavonoids treatments, including high dose (2.52 g·kg^−1^), medium dose (1.26 g·kg^−1^), and low dose (0.63 g·kg^−1^). All the flavonoids extracts were dissolved in 30% glycerol. These treatments were sustained for 10 consecutive days.

### 4.6. Measurements of Wound Healing

To measure (horizontal) wound progression, the skin wound healing rates (WHRs) of each group were measured on day 2, 4, 6, 8, and 10. The wound was covered with transparent film and labeled along the wound edge. Then, the required area was excised and weighed.

WHR = [(W_O_ − W_u_)/W_O_] × 100

W_O_: on day 2, wound area weight; W_u_: Unhealed wound area weight.

### 4.7. Immunohistochemistry

On day 1, 3, 5, 7, and 10, the full-thickness of wound skin and surrounding normal skin was removed from different treated rats. One part of the tissue samples was fixed in 4% paraformaldehyde and placed in paraffin blocks for sectioning and 4-μm sections were sliced to evaluate macrophage contents. The wound tissue sections were stained with anti-mouse CD68 antibody (Boster Biological Technology Co., Ltd., Wuhan, China), followed with biotinylated anti-rabbit IgG-HRP antibody (Boster Biological Technology Co., Ltd., Wuhan, China). Four random views of each slice were observed under a microscope (×40). Then, the integral optical density (IOD) of each view was assessed using the Image-Pro plus 6 software to determinate the CD68 content.

### 4.8. Clinical Chemistry

The frozen full-thickness samples were subsequently homogenized, centrifuged, and the supernatant was isolated for analysis of VEGF, TGF-*β*_1_, and hydroxyproline levels using the VEGF ELISA kit (Nanjing Jiancheng Bioengineering Institute, Nanjing, China), the TGF-*β*_1_ ELISA kit (Nanjing Jiancheng Bioengineering Institute, Nanjing, China), and the hydroxyproline acid hydrolysis kit (Suzhou Comin Biotechnology Co., Ltd., Suzhou, China), respectively.

### 4.9. Statistical Analysis

Results were expressed as means ± standard deviation (SD). Comparisons between the groups were performed using one-way ANOVA followed by least significant difference (LSD) post hoc test with SPSS 22.0; *p* < 0.05 was considered to be statistically significant.

## 5. Conclusions

The present work elucidated that the total flavonoids from *B. balsamifera* could promote wound healing on rats significantly. Twenty-seven compounds were identified from the twenty-nine peaks of total flavonoids of *Blumea balsamifera* (L.) DC., including twenty-one flavonoid analogs, five CQA derivatives and one coumarin. The mechanisms of therapeutic effects were attributed to wound contraction, capillary regeneration, collagen deposition, and re-epithelialization. On day 10, the healing rate of the high dose group was a bit better than the JWH group, and both of them reached nearly 95%. The CD68 levels of all total flavonoids groups reached a peak at day 5 after treatment, suggesting that macrophages were active in the inflammatory stage. This study suggested that total flavonoids of *Blumea balsamifera* (L.) DC. represented an appropriate candidate for skin injuries, and this study also indicated that the flavonoid analogs and CQA derivatives may exert joint synergistic therapeutic effects on skin wound healing rates. Nowadays, with increasing global demands for medicines of botanical origin medicine, this work has opened a window for further exploration of ethnomedicine.

## Supplementary Materials

Supplementary materials can be found at www.mdpi.com/1422-0067/18/12/2766/s1.
