# Supplementary material for: Effects and Mechanisms of Total Flavonoids from Blumea balsamifera (L.) DC. on Skin Wound in Rats
Source: Int J Mol Sci. 2017 Dec 19;18(12):2766. doi: 10.3390/ijms18122766 (PMC5751365; doi:10.3390/ijms18122766)
Supplement: Supplementary file 1 [file ijms-18-02766-s001.pdf]

Supplementary information

Effects and Mechanisms of Total Flavonoids from *Blumea balsamifera* (L.) DC. on Skin Wound in Rats

Yuxin Pang[1,2,3,4], Yan Zhang[1,2], Luqi Huang[1,2*], Luofeng Xu[3,4], Kai Wang[3,4], Dan Wang[3,4], Lingliang Guan[3,4], Yingbo Zhang[3,4], Fulai Yu[3,4], Zhenxia Chen[3,4], Xiaoli Xie[3,4]

Catalog

**Figure S1**	Maximum absorbance wavelength of prepared total flavonoids and standard rutin
**Figure S2**	The calibration plot of rutin
**Figure S4**	UPLC chromatograms at 254 nm of total flavonoids sample in positive ion modes analyzed by UPLC-Q-TOF/MS
**Table S1**	The identified compounds of total flavonoids extract in *Blumea balsamifera* (L.) DC. by UPLC-Q-TOF/MS
**Figure S5**	The chemical structures of identified compounds in total flavonoids preparation by UPLC-Q-TOF/MS
**Figure S3**	The characterization of identified compounds in angelica oil analyzed by using the GC-MS Typical UPLC-Q-TOF/MS base peak intensity (BPI) diagram and chromatogram at 254 nm of total flavonoids extract in negative ion mode and in positive ion mode.
**Table S2**	Effects of total flavonoids in *B. balsamifera* on wound healing rates of rats
**Table S3**	Effects of total flavonoids in *B. balsamifera* on CD68 levels in wound tissue of rats
**Table S4**	Effects of total flavonoids in *B. balsamifera* on VEGF levels in wound tissue of rats
**Table S5**	Effects of total flavonoids in *B. balsamifera* on TGF-β_1 levels in wound tissue of rats
**Table S6**	Effects of total flavonoids in *B. balsamifera* on hydroxyproline levels in wound tissue of rats

Determination of Maximum Absorbance Wavelength

Both the total flavonoids and standard rutin solutions prepared were scanned with UV-Vis spectrophotometer fluctuating from 400 to 800 nm, both of which showed the maximum absorbance wavelength at 500 nm (Figure 1). As a result, the wavelength of 500 nm was selected for measuring.

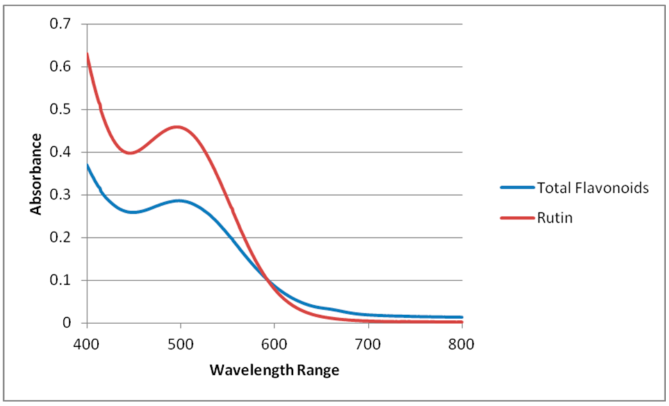

Figure S1. Maximum absorbance wavelength of prepared total flavonoids and standard rutin.

Content of Total Flavonoids

The calibration plot of rutin conducted by ultraviolet-visible spectrometer showed a good linear relationship (Figure S2) with regression equation y = 5.1907x − 0.0098. The fitting degree of curvilinear regression equation was tested by correlation index (R^2) of 0.9996. The absorbance of total flavonoids sample was measured to be 0.8319, accordingly, the content of total flavonoids was deduced to be 81.1%.

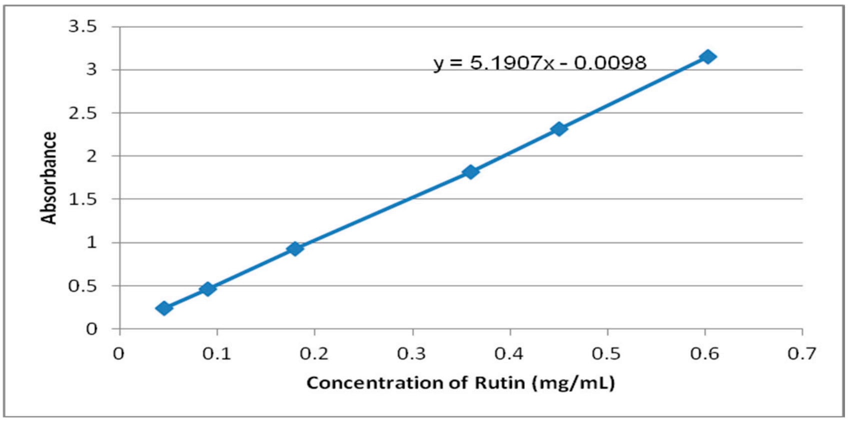

Figure S2. The calibration plot of rutin.

UPLC-Q-TOF/MS Method

The total flavonoids sample was recorded on a Waters Acquity™ Ultra Performance LC system (Waters Corporation, Milford, MA, USA) equipped with a BEH C18 column (100 mm×2.1 mm, 1.7 μm). The flow rate was 0.40 mL/min, the autosampler temperature was 4 °C, and the column compartment was set at 40 °C. The mobile phase was composed of water (A) and acetonitrile (B), with each containing 0.1% formic acid. The gradient system was used as follows: 0–10 min, 5% -50%B; 10–15 min, 50% −90%B; 15–16 min washing with 99% B. The eluent from the column was directed to a Diode Array Detector (DAD) and then to mass a spectrometer with a 0.04 min delay.

A Waters SYNAPT G2 HDMS (Waters Corp., Manchester, UK) was used to carry out the mass spectrometry with an electrospray ionization source (ESI) operating in positive ion mode. The capillary voltages were set at 3.0 and sample cone voltage 40 V and extraction cone voltage 4.0 V, respectively. Using drying gas (nitrogen), the desolvation gas rate was set to 800 L/h at 400 °C, the cone gas rate at 50 L/h, and the source temperature at 100 °C. The scan time and inter scan delay were set to 0.15 and 0.02 s, respectively. Leucine-enkephalin was used as the lock mass in all analyses (m/z 556.2771 for positive ion mode) at a concentration of 0.5 μg/mL with a flow rate of 5 μL/min. Data was collected in centroid mode from 100 m/z to 1500 m/z.

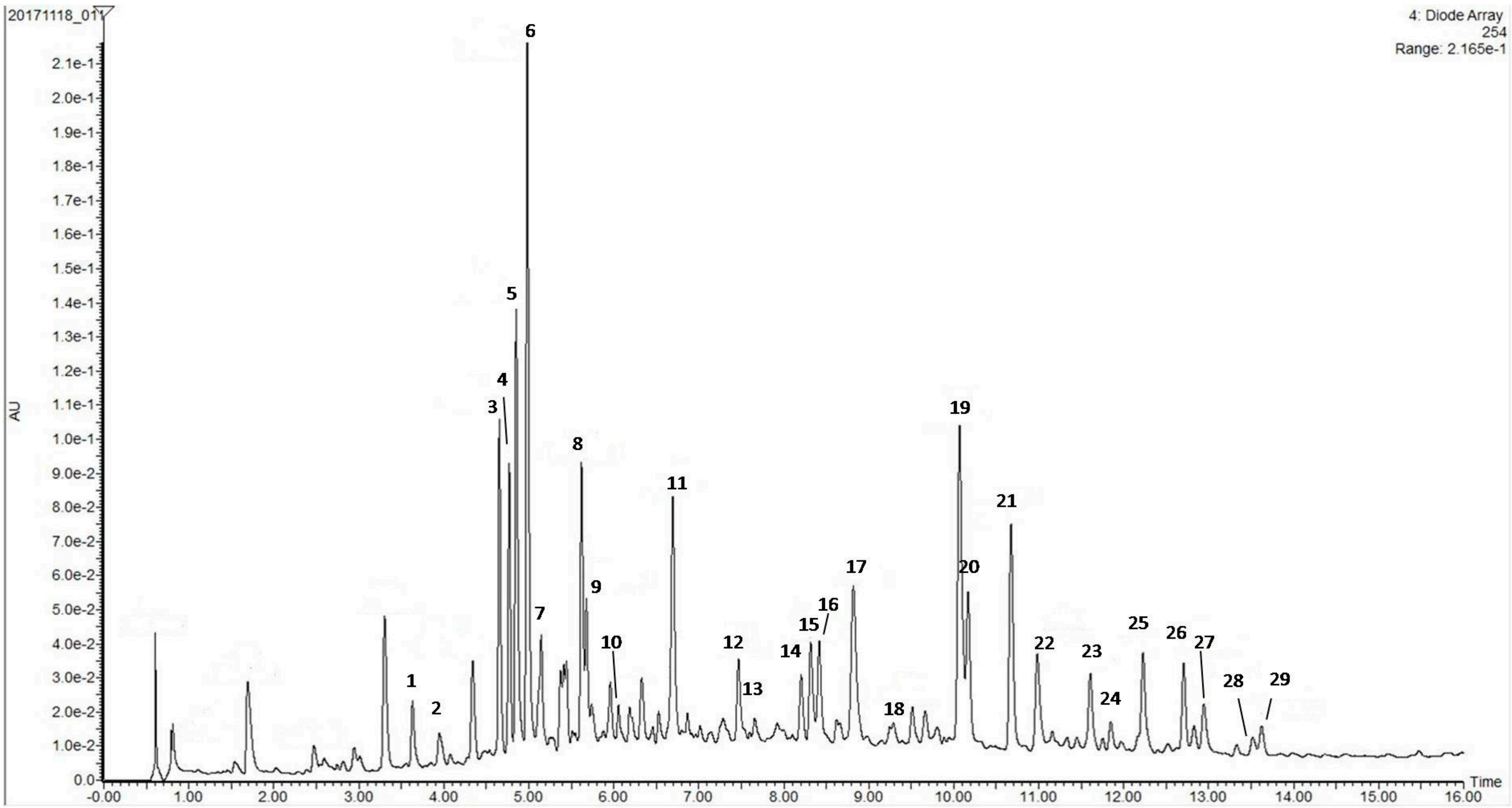

Figure S4. UPLC chromatograms at 254 nm of total flavonoids sample in positive ion modes analyzed by UPLC-Q-TOF/MS/DAD.

Table S1. The identified compounds of total flavonoids extract in *Blumea balsamifera* (L.) DC. by UPLC-Q-TOF/MS

NO.	RT (min)	Identification	Formula	*m/z*	Error (ppm)
1	3.63	4.5-DiCQA	C25H24O12	515.1190	0.0
2	3.95	3.4-DiCQA	C25H24O12	515.1190	0.0
3	4.65	Rutin	C27H30O16	609.1448	1.3
4	4.77	2-(3,4-Dihydroxyphenyl)-5,7-dihydroxy-4-oxo-4H-chromen-3-yl6-O-(6-deoxy-α-L-mannopyranosyl)-D-glucopyranoside	C27H30O16	609.1448	1.3
5	4.85	Hyperoside	C21H20O12	487.0859	1.4
6	4.98	Isoquercitrin	C21H20O12	487.0859	1.4
7	5.14	Myricitrin	C21H20O12	463.0931	0.8
8	5.62	1.3-DiCQA	C25H24O12	515.1190	0.0
9	5.67	3.5-DiCQA	C25H24O12	515.1190	0.0
10	6.05	1,3,5-TriCQA	C34H30O15	679.5042	0.3
11	6.69	3,3',5,7-Tetrahydroxy-4'-methoxyflavanone	C16H14O7	317.0781	2.8
12	7.46	3',5,5',7-Tetrahydroxyflavanone	C15H12O6	287.0758	3.1
13	7.65	Luteolin	C15H10O6	285.0543	2.3
14	8.20	Chrysoeriol	C16H12O6	299.0750	4.8
15	8.31	3,3',4',5-Tetrahydroxy-7-methoxyflavone	C16H12O7	317.0634	2.5
16	8.41	Kaempferide	C16H12O6	301.0724	3.9
17	8.81	Hydranngetin	C10H8O4	193.0521	1.1
18	9.28	Diosmetin	C16H12O6	301.0724	3.9
19	10.06	4',5,7-Trihydroxy-3,3'-dimethoxyflavone	C17H14O7	331.0834	4.8
20	10.16	3,5,4'-Trihydroxy-3',7-dimethoxyflavone	C17H14O7	331.0834	4.8
21	10.66	Blumeatin	C16H14O6	303.0876	2.3
22	10.97	Luteolin-7-methyl-ether	C16H12O6	301.0724	3.9
23	11.60	3,3',5-Trihydroxy-4',7-dimethoxyflavone	C17H14O7	331.0834	4.8
24	11.84	4',5-Dihydroxy-3,3',7-trimethoxyflavone	C18H16O7	345.0978	1.2
25	12.23	Unidentified	C28H24O4	425.1740	3.1
26	12.71	Unidentified	C28H24O4	425.1740	3.1
27	12.94	3,5,7-Trihydroxy-3',4'-dimethoxyflavone	C17H14O7	331.0834	4.8
28	13.51	Ayanin	C18H16O7	345.0978	1.2
29	13.63	5,7-Dihydroxy-3,3',4',-trimethoxyflavone	C18H16O7	345.0978	1.2

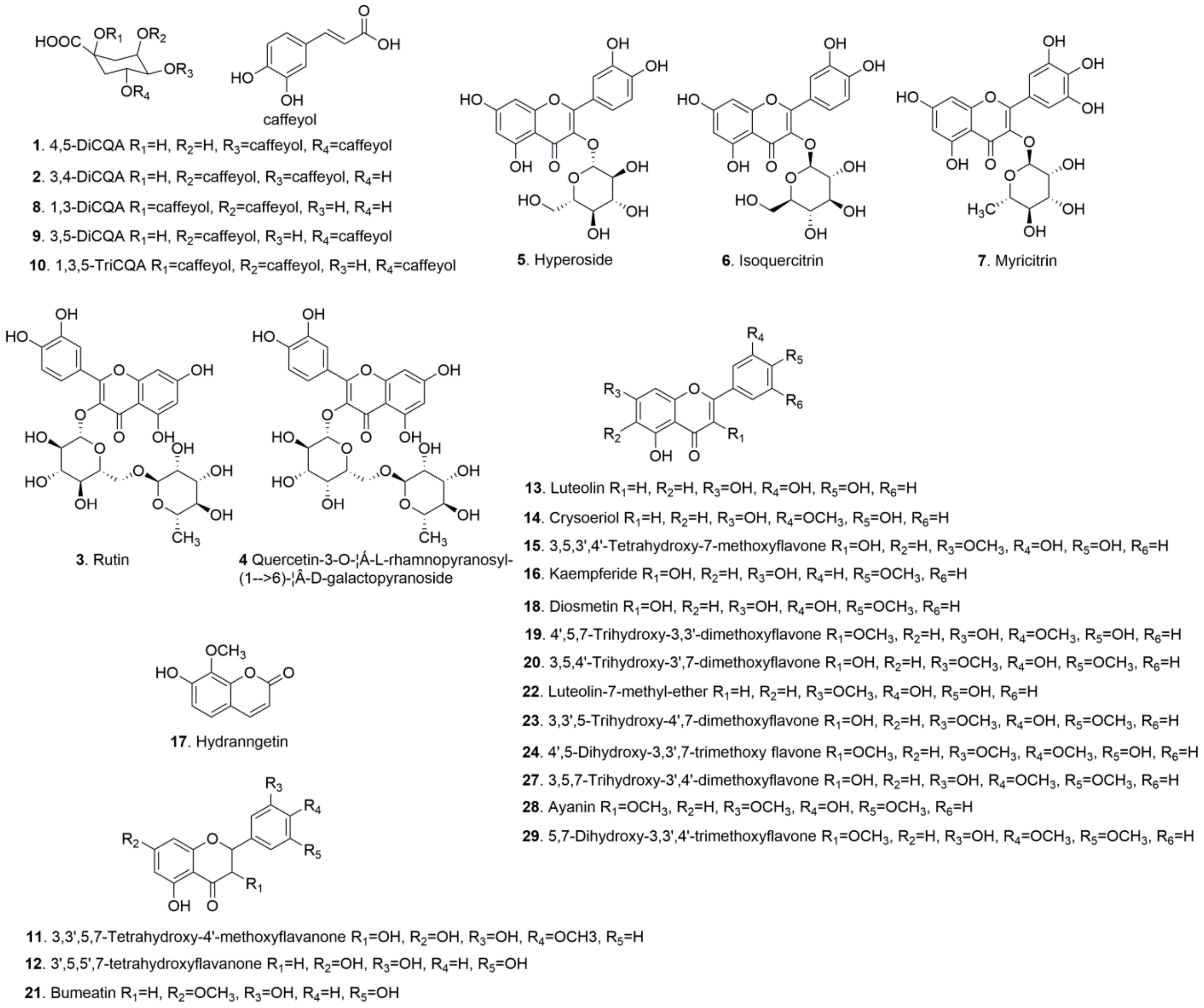

Figure S5. the chemical structures of identified compounds in total flavonoids preparation by UPLC-Q-TOF/MS.

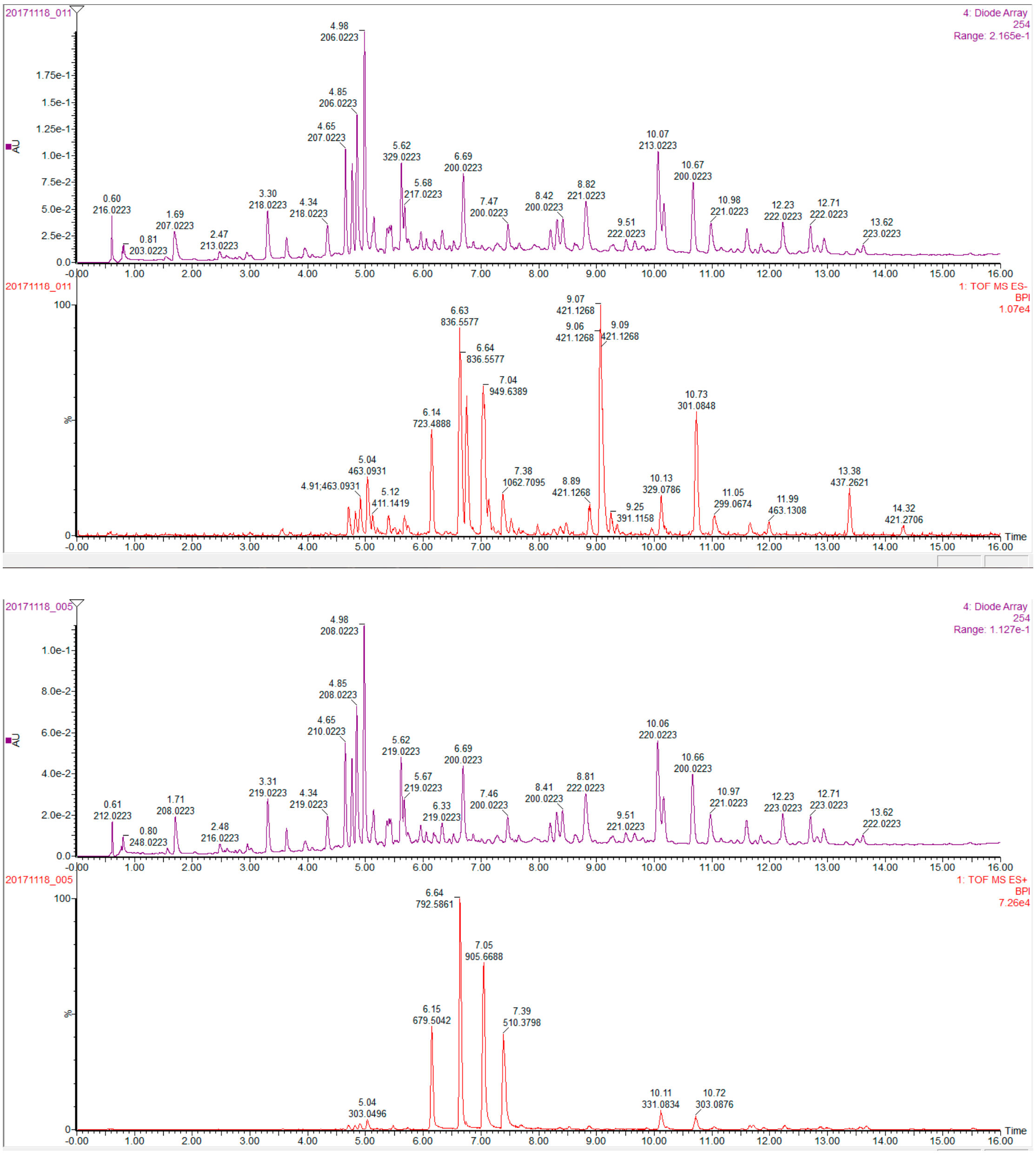

Figure S3. Typical UPLC-Q-TOF/MS base peak intensity (BPI) diagram and chromatogram at 254 nm of total flavonoids extract in negative ion mode and in positive ion mode.

Table S2. Effects of total flavonoids in *B. balsamifera* on wound healing rates of rats.

Groups	Rates of wound healing (%)			
	4 d	6 d	8 d	10 d
High Dose	38.46 ± 0.065 △	53.29 ± 0.050△	81.21 ± 0.054*	95.31 ± 0.062*
Medium Dose	35.75 ± 0.041 △	40.86 ± 0.087	76.15 ± 0.069	88.47 ± 0.061
Low Dose	33.63 ± 0.070 △	39.14 ± 0.065	72.81 ± 0.084	84.02 ± 0.086
Model	22.01 ± 0.088	38.22 ± 0.065	70.25 ± 0.048	82.25 ± 0.775
Positive Control (JWH)	38.03 ± 0.045 △	53.24 ± 0.067△	79.70 ± 0.133*	93.71 ± 0.058*

Note: $^{*}P < 0.05$, $^{\triangle}P < 0.01$

Table S3. Effects of total flavonoids in *B. balsamifera* on CD68 levels in wound tissue of rats.

Groups	IOD ($\times 10^4$)			
	3 d	5 d	7 d	10 d
High Dose	1.87 ± 0.33*	3.34 ± 0.46△	2.64 ± 0.37△	1.31 ± 0.09
Medium Dose	1.68 ± 0.31	2.77 ± 0.53△	2.31 ± 0.46*	1.28 ± 0.04
Low Dose	1.37 ± 0.19	2.40 ± 0.31*	2.00 ± 0.19	1.18 ± 0.08
Model	1.19 ± 0.03	1.67 ± 0.23	1.40 ± 0.17	1.17 ± 0.08
Positive Control (JWH)	1.29 ± 0.29	2.25 ± 0.22	1.78 ± 0.29	1.26 ± 0.12

Note: $^{*}P < 0.05$, $^{\triangle}P < 0.01$

Table S4. Effects of total flavonoids in *B. balsamifera* on VEGF levels in wound tissue of rats.

Groups	Contents of VEGF (ng/g)				
	1 d	3 d	5 d	7 d	10 d
High Dose	107.62 ± 11.07	180.55 ± 16.01*	236.10 ± 27.93*	205.41 ± 16.91	131.59 ± 29.81
Medium Dose	97.69 ± 9.02	161.71 ± 21.93	210.9 ± 9.16	183.81 ± 15.33	141.28 ± 9.25
Low Dose	95.98 ± 21.34	137.66 ± 21.46	163.81 ± 30.96	184.72 ± 22.51	155.22 ± 9.03
Model	96.47 ± 25.49	119.73 ± 11.42	151.34 ± 19.50	152.09 ± 11.69	153.98 ± 13.63
Positive Control (JWH)	108.86 ± 9.25	175.41 ± 24.82*	235.49 ± 37.58*	191.85 ± 40.72	132.45 ± 29.47

Note: $^{*}P < 0.05$, $^{\triangle}P < 0.01$

Table S5. Effects of total flavonoids in *B. balsamifera* on TGF-β_1 levels in wound tissue of rats.

Groups	Contents of TGF-β (ng/g)				
	1 d	3 d	5 d	7 d	10 d
High Dose	17.58 ± 0.58	36.71 ± 1.98*	35.96 ± 1.90*	28.23 ± 1.81*	17.94 ± 1.15
Medium Dose	15.84 ± 1.45	33.11 ± 2.58	34.27 ± 2.88*	26.26 ± 0.50*	18.74 ± 2.13
Low Dose	15.83 ± 0.81	30.98 ± 3.02	30.90 ± 0.94	25.65 ± 0.29*	19.89 ± 1.96
Model	15.06 ± 1.23	25.24 ± 2.37	25.62 ± 0.87	22.41 ± 0.56	20.48 ± 1.49
Positive Control (JWH)	17.61 ± 0.84	38.59 ± 3.96*	36.65 ± 0.52*	27.74 ± 1.03*	17.59 ± 0.39

Note: $*P < 0.05$, $\triangle P < 0.01$

Table S6. Effects of total flavonoids in *B. balsamifera* on hydroxyproline levels in wound tissue of rats**.**

Groups	Contents of hydroxyproline（mg/g）				
	1 d	3 d	5 d	7 d	10 d
High Dose	0.387 ± 0.056	0.423 ± 0.035*	0.572 ± 0.06△	0.701 ± 0.04△	0.795 ± 0.016*
Medium Dose	0.361 ± 0.034	0.409 ± 0.023	0.537 ± 0.054*	0.657 ± 0.036*	0.736 ± 0.035
Low Dose	0.345 ± 0.149	0.402 ± 0.008	0.496 ± 0.042	0.621 ± 0.033	0.692 ± 0.045
Model	0.339 ± 0.065	0.380 ± 0.021	0.449 ± 0.033	0.574 ± 0.021	0.655 ± 0.046
Positive Control (JWH)	0.388 ± 0.098	0.428 ±0.021*	0.578 ± 0.03△	0.707 ± 0.07△	0.793 ± 0.09△

Note: $^{*}P < 0.05$, $^{\triangle}P < 0.01$
